# Genetic variation within a species of parasitic nematode, *Skrjabingylus chitwoodorum*, in skunks

**DOI:** 10.21307/jofnem-2021-005

**Published:** 2021-02-17

**Authors:** Allie N. Denham, Malorri R. Hughes, Robert C. Dowler, Nicholas J. Negovetich, Loren K. Ammerman

**Affiliations:** 1 Department of Biology, Angelo State University, 2601 W. Ave N, San Angelo, Texas, 76909; 2 Department of Biology, Portland State University, 1825 SW Broadway, Portland, Oregon, 97201

**Keywords:** COI, Genetics, *Mephitis*, Nemata, Sinus roundworm, *Spilogale*, Systematics, Texas

## Abstract

Carnivores in the families Mustelidae and Mephitidae are essential hosts for the cranial roundworm genus *Skrjabingylus*. A high prevalence of *Skrjabingylus chitwoodorum* has been observed in the striped skunk, *Mephitis mephitis*. Genetic barcoding studies of other nematodes have successfully used the cytochrome oxidase I (COI) mitochondrial gene to analyze genetic variation and divergence. We tested the hypothesis that low population structuring occurs within *S. chitwoodorum* because *M. mephitis* is widespread across much of North America and has high levels of gene flow. We extracted DNA from 38 samples of *Skrjabingylus* removed from the sinuses of *M. mephitis* and one from the plains spotted skunk, *Spilogale putorius interrupta*, for amplification and sequencing of COI. Analysis of 492 base pairs confirmed all samples were *S. chitwoodorum* and showed low genetic divergence (1.0%) within Texas, but high haplotype diversity. Supporting our hypothesis, no obvious divergent lineages based on geographic location were recovered within the samples based on Maximum Likelihood analysis and median joining haplotype network analysis. In fact, samples of *Skrjabingylus* from New York and South Dakota showed little difference compared with samples from Texas.

The endoparasitic nematode genus, *Skrjabingylus,* is known to target carnivore hosts in the family Mustelidae, such as weasels, minks, martens, and otters (Santi and Parker, 2012). Along with mustelids, *Skrjabingylus* also occurs often in skunks, family Mephitidae (Kirkland and Kirkland, 1983). The adult parasitic nematode resides primarily within the frontal nasal sinuses of the definitive host. Six species have been described and most are host-specific, with *Skrjabingylus nasicola* Leukart, 1842 infecting definitive hosts within the members of the genus *Mustela* (weasels, polecats, and stoats), and the genus *Neovison* (minks; Hawkins et al., 2010). *Skrjabingylus chitwoodorum* (Hill, 1939) is found in skunk genera *Mephitis* and *Spilogale*, *Skrjabingylus petrowi *Bagenanow and Petrov, 1941 and *Skrjabingylus ryjikovi* Kontrimavichus, 1961 in the genus *Martes* (Heddergott et al., 2015), *Skrjabingylus lutrae* in river otters (Lankester and Crichton, 1972), and the newest species to be described, *Skrjabingylus santaceciliae*, is found in *Mephitis macroura* (Carreno et al., 2005).

Morphology has been the main focus of research studies within the genus *Skrjabingylus* as little genetic work has been done thus far. Of the six known species, only four occur in the Americas: *S. nasicola*, *S. lutrae*, *S. santaceciliae*, and *S. chitwoodorum* (Carreno et al., 2005; Lankester, 1983). The four species are distinguished through body size, length of the male spicules, a distinctive shape of the distal tip of the spicule, and the host of the parasite (Lankester, 1983). The length of spicules is a common way to differentiate *Skrjabingylus* in the Americas because they are each so distinct from one another; *S. nasicola* ranges from 180 to 232 µm (Lankester, 1983), *S. lutrae* ranges from 239 to 275 µm (Lankester and Crichton, 1972), *S. santaceciliae* ranges from 385 to 428 µm (Carreno et al., 2005), and *S. chitwoodorum* having known ranges of 540 to 890 µm (Hill, 1939; Webster, 1965).

The lifecycle of *Skrjabingylus* species are all very similar (Fig. 1) and the larval stages have similar sizes and structures (Santi and Parker, 2012). *Skrjabingylus* begins as first-stage larvae in gastropods, mollusks, or fish that can act as intermediate hosts. The intermediate host holds the J_1_ larvae that develop to J_3_ until the host is either eaten directly by the definitive host or a paratenic host. A paratenic host acts as a bridge to the definitive host and little to no development will occur in this host. Once in the definitive host, the third-stage larvae enter the gut and molt twice to become J_5_ larvae. Around four to five days post-ingestion, the worms burrow through the intestinal wall, and then migrate up localized nerves for six days until they reach the sinuses for maturation (Lankester and Anderson, 1971). The cycle repeats when the skunk passes J_1_ larvae (all species except *S. lutrae* are viviparous) through the gastrointestinal system and then gastropods or other intermediate hosts ingest them in the fecal matter (Hansson, 1967; Santi and Parker, 2012; Fig. 1).

**Figure 1:**
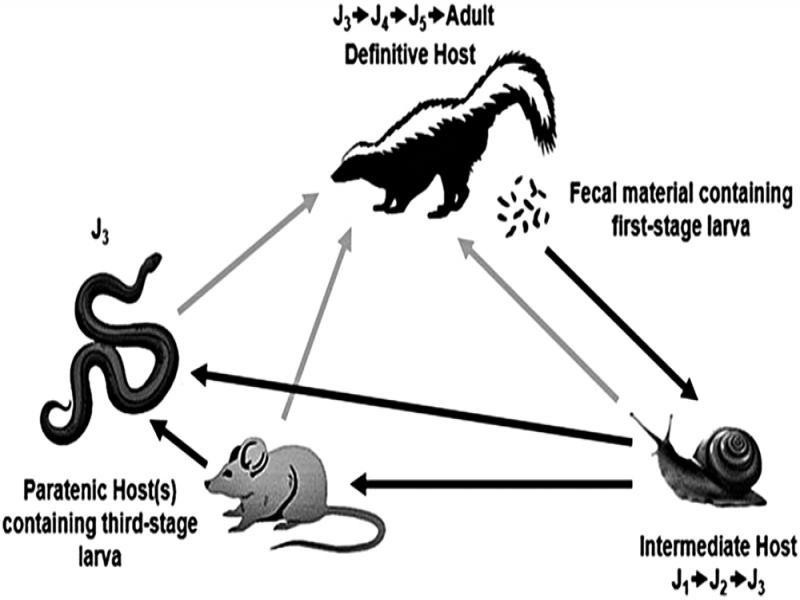
Life cycle of *Skrjabingylus chitwoodorum* in *Mephitis mephitis.* Large gray arrows correspond to the movement of *Skrjabingylus* to an intermediate or paratenic host. Large black arrows correspond to the movement to the definitive host. Smaller arrows correspond to a molt occurring and the larva progressing to the next juvenile phase.

There is some evidence of *Skrjabingylus* creating meningitis during the migration to the olfactory nerves, which also can create severe blockages and therefore not permit infection in the sinus (Lankester and Anderson, 1971). Skunks also have been seen to display obvious neurological disturbances after a mere 13 days post-infection (Lankester, 1970). Lankester reported certain skunks experiencing loss of motor coordination and lethargy. These findings are similar to numerous observations of “dancing” mustelids or mephitids experiencing some sort of episode or epileptic crisis (Debrot and Mermod, 1980; King, 1989; Lewis, 1967).

*Skrjabingylus chitwoodorum* has shown a high prevalence in Texas within the host species, *M. mephitis.* Hughes et al. (2018) examined 595 striped skunks from Texas and extracted nematodes directly from the sinuses of 48.7% of them. The authors concluded that *S. chitwoodorum* had a bias for the left sinus and also that ecoregion and precipitation was not a determining factor of prevalence. However, Higdon and Gompper (2020) concluded after examining 578 skulls of *Spilogale putorius* that the prevalence and intensity of *S. chitwoodorum* was correlated with precipitation, and they concluded that higher levels of precipitation most likely caused an increase in mollusks that act as intermediate hosts. Underlying genetic differences that allow the parasite to be successful across a variety of habitats could be present, but this has not been examined.

Previous research reported high gene flow in the host of *S. chitwoodorum*, *M. mephitis,* in an urban population in Texas (Brashear et al., 2015), as well as on a larger scale across 22,000 square kilometers in Canada, regardless of geographic barriers (Talbot et al., 2012). We expected *Skrjabingylus* to have a similar pattern to *M. mephitis* and exhibit little genetic structuring across the sample area. Generally, the more host-specific the parasite is, a greater similarity in host and parasite phylogenies can be exhibited (Dougherty, 1949). In contrast, patterns of mitochondrial sequence evolution in *S. chitwoodorum* could reveal undescribed variation and/or barriers to gene flow. COI rather than nuclear DNA (such as ribosomal genes) was selected to study the population genetics of the nematode because this gene has been used successfully in several studies (i.e. Lazarova et al., 2006; Prosser et al., 2013). Derycke et al. (2010) used the COI barcoding technique in 41 free-living marine nematode species to discriminate one morphological species from another. They were unable to differentiate only two out of 41 species (Derycke et al., 2010). This meant that COI sequence showed reasonable variability among species and could likely differentiate lineages of *S. chitwoodorum* from one another. Because little genetic work has been performed, especially at the intraspecific level, on *S. chitwoodorum,* this study tested the hypothesis that there is a single lineage of *Skrjabingylus* in *M. mephitis* in Texas. The alternative hypothesis was that the gene sequences would vary and recover a pattern of substructuring of lineages of *Skrjabingylus*. The objective of this study was to use COI sequences to determine the level of genetic variation within *S. chitwoodorum*, to describe the patterns of variation with respect to geographical sampling locality, and to determine the phylogenetic relationship of *S. chitwoodorum* to other *Skrjabingylus* species.

## Material and methods

**Figure 2:**
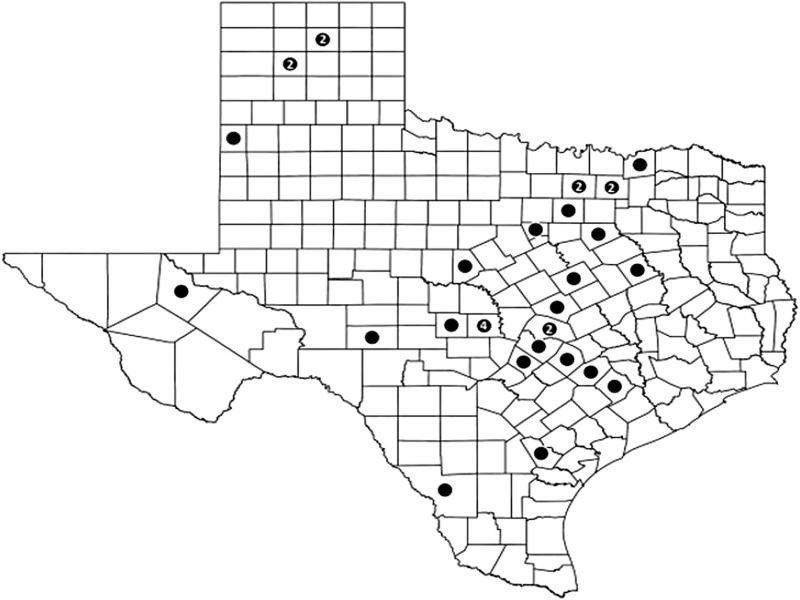
Texas map showing the 25 counties represented in the analysis of *Skrjabingylus* within *Mephitis mephitis* hosts. Sample size included if greater than one.

A total of 19 *Skrjabingylus* samples that were reported in Hughes et al. (2018) were used for the present study. Only samples that were preserved in ethanol or were frozen in the Angelo State Natural History Collections (ASNHC) were requested for our DNA analysis. Most of these originated from the rabies lab at Texas Department of State Health Services in Austin. Additional skulls of rabies-negative skunks were deposited in the ASNHC and were screened for *Skrjabingylus* when the skulls were prepared as voucher specimens. Samples were identified by the tissue number (ASK number) of their host. In total, we obtained samples of *Skrjabingylus* from 33 *M. mephitis* from 25 Texas counties (Fig. 2). Sample sizes from each county ranged from one to four skunks. This allowed a broad coverage in Texas and five additional specimens were acquired from New York (3) and South Dakota (2). We obtained one additional *Skrjabingylus* sample from *Spilogale putorius interrupta* (Eastern Spotted Skunk) from South Dakota from a trapper-salvaged animal, and New York samples were provided by New York State Department of Health Griffin Laboratory. These additional six samples were as follows: three from Brule County, South Dakota, including the *Spilogale* sample; one from Clinton County, New York; and two from Franklin County, New York. Host voucher specimens were deposited in the Angelo State Natural History Collections and representative vouchers of *S. chitwoodorum* were deposited at the University of Nebraska-Lincoln’s Harold W. Manter Laboratory of Parasitology (HWML-111593 to 111597).

To amplify a 661-base pair (bp) fragment of COI we used primers NemF2_t1: ARAGATCTAATCATAAAGATATYGG and NemR2_t1: AWACYTCWGGRTGMCCAAAAAAYCA (Prosser et al., 2013). DNA was extracted from individual *S. chitwoodorum* with the Qiagen DNeasy Blood and Tissue Kit and the following methodology: a sample of tissue was added to 180 µL ATL buffer and 20 µL proteinase K. A sample was vortexed and incubated for 48 hrs (56°C) to lyse completely. After 24 hr of incubation, we added 20 µL proteinase K. After 48 hr, 200 µL buffer AL was added and the tube was vortexed. The samples were then incubated 70°C for 5.5 hr. After 5.5 hr, 200 µL of ethanol was added and vortexed, followed by the contents being transferred to a spin column and the steps in the manufacturer’s protocol were followed. Lastly, the elution occurred with 50 µL AE elution buffer. If the sample was preserved in ethanol, the worm was soaked three times in 1X PBS (phosphate buffered saline) for 10 min each before DNA extraction occurred in order to rehydrate the sample. After extraction occurred, purity and concentration were measured using a NanoDrop Lite Spectrophotometer.

The final reaction conditions used to amplify COI were as follows: 0.25 to 2 µL template DNA ranging from 1.2 to 110 ng, 0.08 U *Taq* polymerase (New England Biolabs, Ipswich, MA), 0.8 mM of each dNTP (Thermo Fisher Sci., Waltham, MA), 1X standard *Taq* reaction buffer (New England Biolabs, Ipswich, MA), 2 mM MgCl_2_ (New England BioLabs, Ipswich, MA), 0.16 µM of each forward and reverse primers (Alpha DNA, Montreal, Quebec, Canada), and RNase-free water as needed to reach a final volume of 12.5 µL. The *r* reactions were amplified using the following thermalcycler profile modified from Heddergott et al. (2015): a denaturing step of 94°C for 1 min, followed by 40 cycles of 94°C for 40 sec, annealing at 51°C for 40 sec, 72°C for 1 min, and a final extension of 72°C for 5 min. Products were analyzed and verified using gel electrophoresis and a FastRuler middle range ladder (Thermo Fisher Sci., Waltham, MA) to determine gene amplification. Quantification of PCR product was obtained using a fluorometric Qubit HS Assay kit (Thermo Fisher Sci., Waltham, MA). Samples with a minimum of 80 ng were then purified with ExoSAP-IT Express PCR Product Cleaning Reagent (Thermo Fisher Sci., Waltham, MA) following the manufacturer’s protocol. The purified samples were then shipped to the Texas A&M Corpus Christi Genomic Core Sequencing Lab for Sanger sequencing of both DNA strands.

The sequences were analyzed using Sequencher^®^ version 5.4.6 DNA sequence analysis software (Gene Codes Corporation, Ann Arbor, MI USA) to assemble contigs from forward and reverse sequences and create consensus sequences for each individual. After confirming that each sequence translated to protein correctly, we exported the sequences and then aligned them in MEGA X (Kumar et al., 2018) using the MUSCLE algorithm. We submitted sequences to the NIH GenBank database (accession numbers MT454072-MT454110). Phylogenetic analysis among species of *Skrjabingylus* was conducted with MEGA X (Kumar et al., 2018) using the gene sequences of *S. chitwoodorum* that we generated and two other species of *Skrjabingylus* were downloaded from GenBank, *S. petrowi* (KP724692-KP724694) and *S. nasicola* (KP724695-KP724696). A phylogenetic tree was created on MEGA X using Maximum Likelihood criteria and the best-fitting DNA model, Hasegawa-Kishino-Yano + G (HKY + G). Bootstrap analysis (1,000 replicates) was conducted to evaluate the support of each branch. Genetic divergence was calculated based on COI both within *S. chitwoodorum* (among Texas counties) and between Texas and samples from New York and South Dakota. We also calculated divergence among the three *Skrjabingylus* species using Kimura-2 parameter (K2P) for pairwise-distance computation in MEGA X (Kumar et al., 2018). K2P was used for genetic divergence analysis because it was the model that had the closest substitution rate and pattern to HKY + G. A median joining haplotype network analysis was performed using PopArt to identify haplotypes and further show possible intraspecific patterns (Leigh and Bryant, 2015) across the area sampled.

## Results

**Figure 3:**
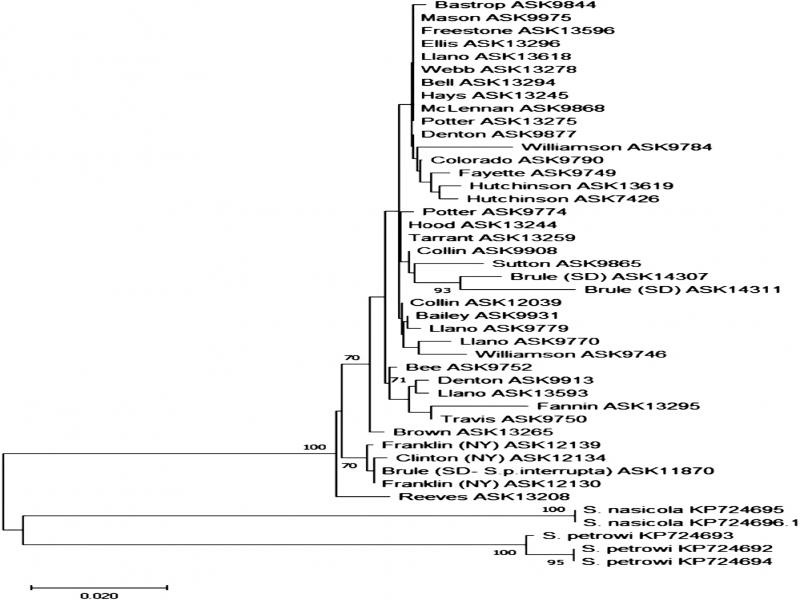
Maximum likelihood phylogenetic tree of 492 base pair fragment of the cytochrome oxidase I gene for 44 samples of *Skrjabingylus.* Maximum likelihood analysis was performed using the best-fitting model, Hasegawa-Kishino-Yano of DNA substitution with Gamma distribution. Bootstrap values are based on 1000 replicates and values ≥70 are shown on branches. Number with prefix ASK identifies the specific host from which the sample was collected. Prefix KP is a Genbank accession number.

No significant pattern was seen between amplification of COI and correlation to purity ratio and/or concentration of template DNA. COI sequences were obtained from 39 samples of *Skrjabingylus* from 38 *M. mephitis* hosts and 1 *S. p. interrupta* host, representing areas of Texas (33), New York (3), and South Dakota (2). Maximum likelihood analysis of the fragment of COI composed of 492 base pairs recovered three distinct lineages: *S. petrowi*, *S. nasicola*, and a lineage comprising all *S. chitwoodorum* (Fig. 3). Bootstrap analysis results using 1,000 replicates supported a distinct lineage of *S. chitwoodorum* that was divergent from the other two species of *Skrjabingylus*. Among all three species, 125 polymorphic sites were documented. *Skrjabingylus chitwoodorum* was 14.7% divergent from the outgroup taxon *S. petrowi* and 14.4% divergent from *S. nasicola.* A 15.9% difference was observed between *S. nasicola* and *S. petrowi* (Table 1).

**Table 1.** Average Kimura-2 parameter distance among outgroup taxa *Skrjabingylus nasicola* and *Skrjabingylus petrowi* and populations of *Skrjabingylus chitwoodorum* from *Mephitis mephitis* in various states based on 492 bases of COI.

	*S. nasicola*	*S. petrowi*	*S. chitwoodorum*	Texas	South Dakota	New York
*S. nasicola* (2)	*0.000*					
*S. petrowi* (3)	0.159	*0.005*				
*S. chitwoodorum* (39)	0.146	0.142	*0.013*			
Texas (33)	0.144	0.147	–	*0.010*		
South Dakota (3)^a^	0.159	0.157	–	0.023	*0.030*	
New York (3)	0.140	0.134	–	0.018	0.023	*0.002*

**Notes:** Sample sizes are listed in parentheses. ^a^One of the samples from South Dakota was from *Spilogale putorius interrupta.*

Within *S. chitwoodorum* samples, there was no obvious pattern of close geographic relationships other than a branch of four out-of-state samples, three from New York, and one from South Dakota, that were placed at the base of the tree and had bootstrap support of 70% (Fig. 3). The sample of *S. chitwoodorum* from *Spilogale* (ASK11870) collected in South Dakota clustered closely with other samples collected from *M. mephitis*. However, the other two samples from South Dakota striped skunks were sister taxa (supported by a 93% bootstrap value) and clustered with a sinus worm from Sutton County, Texas. For the most part, among the samples from Texas there were no well-supported lineages that linked samples from the same county of collection. In total, 58 polymorphic sites occurred within the 39 *S. chitwoodorum* samples included in this study, 20 of which were parsimony-informative and 38 that were singleton sites. The overall average variation within the 33 Texas samples was 1.00% genetic divergence (Table 1). The genetic diversity slightly increased when the six out-of-state samples were added into the analysis, showing a 1.29% average divergence. The highest divergence within Texas samples was 3.78% between Fannin (ASK13296) and Williamson (ASK9784) counties. When comparing Texas samples to the six out-of-state samples, the highest variation was 5.24% between Fannin (ASK13296) County in Texas and Brule (ASK14311) County in South Dakota.

**Figure 4:**
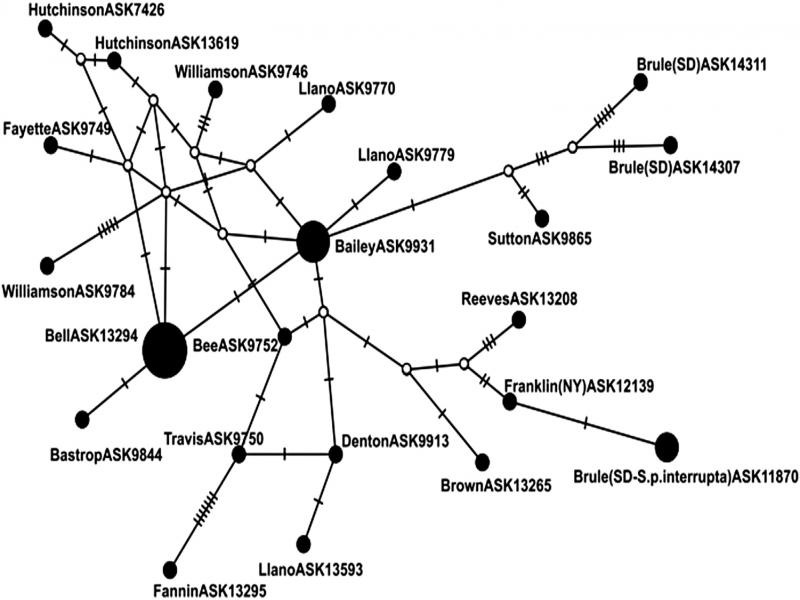
Median joining network showing the relationships among haplotypes of 44 samples of *Skrjabingylus chitwoodorum* from hosts *Mephitis mephitis* and *Spilogale putorius interrupta* using COI mtDNA. Sizes of solid black circles correlate to shared haplotypes among multiple counties. Small open circles represent hypothetical haplotypes and ticks on branches represent number of mutational steps.

In total, there were 22 COI haplotypes in *S. chitwoodorum* with three haplotypes being shared by two or more samples (Fig. 4). The Bell County (ASK13294) haplotype was the most common and was shared with 10 other samples. Bailey County (ASK9931) shared a haplotype with five other samples from Texas (additional details available in Denham, 2020). Interestingly, the one sample of *Skrjabingylus* from the host species *Spilogale* in Brule County (ASK11870) of South Dakota shared a haplotype with two other counties, Clinton and Franklin County in New York (ASK12134 and ASK12130). Nucleotide diversity, represented by π, was 3.06 × 10^6^. Mutational steps ranged between one to seven changes with the highest occurring between Travis County (ASK9750) and Fannin County (ASK13295).

## Discussion

This study used genetic data to show that *S. chitwoodorum* is a distinct lineage present in the three US states from which samples were acquired (Texas, South Dakota, and New York). In addition, there was a low level of divergence in COI, even if samples were separated by long distances. Lastly, the analysis showed a lack of a distinct pattern of genetic lineages that mirror geography, which suggests high levels of gene flow in both host and parasite. Thus, these data supported our hypothesis that *S. chitwoodorum* exhibits little genetic structuring across the sample area.

Each of our 39 samples clustered together in their own clade clearly separate from other *Skrjabingylus* species. A 14.2 to 14.6% genetic difference of *S. chitwoodorum* from both outgroup species is similar to that of Heddergott et al. (2015) who reported 12.5% sequence divergence between *S. nasicola* and *S. petrowi* for COI. *Skrjabingylus chitwoodorum* sequences from across the country remained well conserved as seen by an average of 1.29% genetic divergence and shared haplotypes from distant sites of occurrence. This low level of genetic structuring is consistent with studies that found high gene flow in their *Mephitis* host (Brashear et al., 2015; Talbot et al., 2012). Therefore, the movements of the definitive host or even the intermediate or paratenic host might be influencing the lack of genetic structuring we observed in *S. chitwoodorum*. Other genetic studies have found similar results with parasites experiencing the same evolutionary patterns as their hosts. One example is *Baylisascaris schroederi*, a nematode found in the stomach of wild giant pandas in China (Zhou et al., 2013). Similar to our results, Zhou et al. (2013) reported low divergence (2.80%) in *B*. *schroederi* and a significant, high level of gene flow in 44 samples from genetically distinct giant panda populations from three mountain ranges using a barcoding method on the cytochrome b mitochondrial gene. A separate study on the plant-parasitic nematode, *Longidorus poessneckensis,* also showed similar findings (Kornobis et al., 2017). After an analysis on 16 populations, COI revealed low variability (0-2.4%) while nicotinamide dehydrogenase subunit 4 (*nad4*) showed a higher variability (0-7.6%). These contrasting results show that the particular loci being analyzed are important in determining intraspecific variability. More loci, both mitochondrial and nuclear, should be analyzed in future studies to have a further understanding of intraspecific variation within *S. chitwoodorum.* A full nuclear genome study could also be beneficial as no known full genome projects have occurred with *Skrjabingylus*.

One sample of *Skrjabingylus* (ASK11870) from the host species *Spilogale putorius* in Brule County of South Dakota shared a haplotype with two other counties, Clinton and Franklin County in New York (ASK12134 and ASK12130). It is important to note that the *Spilogale* sample was processed (DNA extraction and PCR) separately from the *Mephitis* samples to limit chances of contamination. Although found in different skunk genera, *S. chitwoodorum* showed little to no variation and expressed identical mutations with samples separated by approximately 2,050 km from Clinton County in New York. A shared haplotype between two genera of hosts provides opportunity for future research to investigate host-specificity in *S. chitwoodorum* compared to the other species of *Skrjabingylus,* which generally (except *S. nasicola*) reside only within one genus or species (Tumlison and Tumlison, 2019).

Increased sampling across the United States might have provided a better understanding of geographic variation in *S. chitwoodorum*. Additionally, acquiring more samples from the same regions (counties) would have allowed us to perform population-level genetic analyses. Similarly, only one sample from host genus *Spilogale* was present in our study. More samples from the distributional range of this genus in North America are needed to assess potential patterns of variation in spotted skunks. We encourage researchers to preserve endoparasites such as *Skrjabingylus* in ways that facilitate molecular analysis (freezing or storage in ethanol instead of formalin).

We have shown that *S. chitwoodorum* forms a distinct genetic lineage from two other described species of *Skrjabingylus* based on COI. Overall, additional studies are necessary to understand more fully the biodiversity within the genus *Skrjabingylus.*
